# SNHG29 regulates miR-223-3p/CTNND1 axis to promote glioblastoma progression via Wnt/β-catenin signaling pathway

**DOI:** 10.1186/s12935-019-1057-x

**Published:** 2019-12-19

**Authors:** Lizhang Han, Zhonggang Li, Yuquan Jiang, Zheng Jiang, Ling Tang

**Affiliations:** 1 Department of Neurosurgery, Qilu Hospital of Shandong University and Institute of Brain and Brain-Inspired Science, Shandong University, 107 Wenhua West Road, Lixia District, Jinan, 250012 Shandong People’s Republic of China; 2 Shandong Key Laboratory of Brain Function Remodeling, Jinan, People’s Republic of China; 3 Department of Neurosurgery, Linyi People’s Hospital, 27 Jiefang Road East Section, Lanshan District, Linyi, Shandong China; 4 Department of Pediatrics, Jinan Central Hospital Affiliated to Shandong University, Jinan, 250013 People’s Republic of China

**Keywords:** SNHG29, miR-223-3p, CTNND1, Glioblastoma, Wnt/β-catenin signaling pathway

## Abstract

**Background:**

Glioblastoma has been seen as the most common malignancy of brain tumor. Emerging reports has claimed that SNHG29 (LRRC75A-AS1) was involved in several biological processes via modulation of signaling pathway, and served as an malignant facilitatorin osteosarcoma. However, the specific role of SNHG29 in glioblastoma remains unknown.

**Methods:**

RT-qPCR and microarray were operated to measure genes expression. Western blot was performed to examine protein expression. CCK-8 and colony formation assays were used to evaluate cell proliferation. Cell migration was tested by transwell assay. Nuclear-cytoplasmic fractionation was conducted to locate SNHG29. The binding capacity of miR-223-3p to SNHG29 or CTNND1 3′UTR was verified by RIP and luciferase reporter assay.

**Results:**

SNHG29 presented high expression in glioblastoma to boost cell proliferation, migration and EMT process. In addition, miR-223-3p was validated to bind with SNHG29 after prediction and screening. Furthermore, miR-223-3p was proved to be a negative regulator for its target CTNND1. Then, the inhibition on cell proliferation, migration and EMT process resulted from SNHG29 knockdown was recovered by CTNND1 overexpression. At last, the inhibitive impacts on cell proliferation, migration and EMT process of CTNND1 deficiency was abrogated by LiCl.

**Conclusions:**

In conclusion, SNHG29 regulates miR-223-3p/CTNND1 axis to promote glioblastoma progression via Wnt/β-catenin signaling pathway, offering a potential therapeutic point for glioblastoma patients.

## Background

Glioblastoma has been regarded as one of the most common brain malignancy with high mortality [1]. In spite of advances in modern therapies like chemotherapy, radiotherapy and surgery, glioblastoma patients remained to display the poor prognosis with low 5-year survival rate [2–4]. Worse still, the pathogenesis and development of glioblastoma were extremely complicated [5]. Thereby, gene-targeted therapy was supposed to be a relatively effective therapeutic tactic for patients with glioblastoma.

Genome‑wide sequencing has disclosed that less than 2% of genes were capable to encode proteins and more than 98% of the genome was non-coding genes. Long non-coding RNA (lncRNA) was one of non-coding RNAs (ncRNAs) with more than 200 nucleotides in length, which has been identified to play a critical role in diverse biological processes to stimulate diseases or tumors initiation and progression. SNHG8 was reported to directly sponge with miR-663 to regulate the growth, migration, and invasion of colorectal cancer cells [6]. MALAT1 has been confirmed to improve cell cycle progression of pulmonary artery hypertension [7]. Small nucleolar RNA host gene 29 (SNHG29) was also known as LRRC75A-AS1, which has been identified to regulate the development osteosarcoma [8]. Additionally, SNHG29 was proved to be involved in the modulation of vascular calcification [9]. Nevertheless, the biological roles of SNHG29 in glioblastoma remained unknown.

LncRNAs were supposed to regulate gene expression in several mechanisms such as gene imprinting, epigenetic modification and degrading miRNAs. Except those regulatory mechanisms, emerging researches proposed that lncRNAs functioned as a competitive endogenous RNA (ceRNA) by sponging microRNAs (miRNAs) to regulate the expression of target genes. As examples, the regulatory mechanism of HOTAIR in prostate cancer was to sponge miR-152 to increase the expression of FOXR2, modulating proliferation and apoptosis of prostate cancer cells [10]. Long non-coding RNA PVT1 regulate miR-143/HK2 axis to drive tumor progression in gallbladder cancer [11]. However, the regulatory mechanism of SNHG29 in glioblastoma has not been explored yet.

This research was aimed to explore the biological function and regulatory mechanism of SNHG29 in glioblastoma. We confirmed that SNHG29 regulates miR-223-3p/CTNND1 axis to promote glioblastoma progression via Wnt/β-catenin signaling pathway, implying a potential tactic for the treatment of glioblastoma patients.

## Materials and methods

### Tissue samples and cell lines

The glioblastoma samples (n = 33) and normal brain tissues (n = 33) were obtained from Qilu Hospital of Shandong University. All the specimens were rapidly maintained at − 80 °C. All of these patients have not received other anticancer treatment prior to operation. Informed consents of this research were signed by patients before surgery, and this exploration was approved by the Ethics Committee of normal human astrocyte (NHA) was purchased from Sciencell Research Laboratories (Carlsbad, CA, USA). In addition, human glioblastoma cells including U87, U251, Hs683 and CCD-25Lu were acquired from Chinese Academy of Science Cell Bank (shanghai, China). NHA cells were grown in astrocyte medium, while glioblastoma cells were cultivated in the Dulbecco’s Modified Eagle Medium (DMEM, Gibco, USA) added with 10% fetal bovine serum (FBS). A damp atmosphere containing 5% CO_2_ at 37 °C was suitable for incubation of cell lines.

### Cell transfection

The short hairpin RNA (shRNA) targeting SNHG29 or CTNND1 (sh-SNHG29#1/2/3 or sh-CTNND1#1/2/3) with negative control (sh-NC) was employed to knockdown SNHG29 or CTNND1 expression. Overexpressed miR-233-3p was obtained by transfected with miR-223-3p mimics with negative control (NC mimics). Overexpression vector for CTNND1 was constructed by cloning the full length of CTNND1 into pcDNA3.1 vector, and the empty pcDNA3.1 was taken as control. All the plasmids were synthesized by GenePharma (Shanghai, China) and transfected into glioblastoma cells through using Lipofectamine 2000 (Invitrogen, Carlsbad, CA, USA) according to the manufacturer’s instruction.

### Real-time reverse-transcription polymerase chain reaction (RT-qPCR)

Total RNA extraction from the tissues and cells was made with Trizol reagent (Life Technologies Corporation, Carlsbad, CA, USA). The expression of SNHG29, CTNND1, FOXO1, CTNNB1, cyclin D1, c-myc and their internal control GAPDH in glioblastoma tissues, cells as well as corresponding controls was detected by SYBR Prime-Script RT-PCR Kit (TakaraBio, Japan). The expression of miR-223-3p and its endogenous control U6 was examined by TaqMan MicroRNA Reverse Transcription kit and TaqMan Universal Master Mix II (Applied Biosystems, Foster City, CA, USA). Relative expression values were measured and calculated through using 2^−∆∆Ct^ method.

### Cell proliferation assay

The Cell Counting Kit-8 (CCK-8) assay and colony formation assay were adopted to assess cell proliferation. For CCK-8 assay, U87 and U251 cells (1000 cells/well) transfected with sh-NC or SNHG29-specific shRNAs were cultivated in 96-well plates. 10 µl of CCK-8 solvent was supplemented to each well at five time-points. The absorbance at the wavelength of 450 nm was assessed using a microplate reader.

For colony formation assays, U87 and U251cells (250) were cultured for 14 days after being seeded in each well of six-well plates. To calculate colonies, 4% paraformaldehyde and crystal violet were separately used for fixation and staining.

### Nuclear-cytoplasmic fractionation

The cytoplasmic or nuclear SNHG29 was segregated with the application of a PARIS kit (Life Technologies, MA, USA). Briefly, collected U87 and U251 cells were lysed on ice. The supernatant was harvested after centrifugation. The extracted RNAs and two controls (GAPDH for cytoplasm, U6 for nucleus) were tested by RT-qPCR, respectively.

### Transwell assay

SNHG29-downregulated U87 and U251 cells were placed in the upper chamber. Afterwards, the lower chamber was supplemented with 600 µl of DMEM containing 10% FBS (Hyclone, Shanghai, China). After incubated for 24 h, cells on the lower chamber were subjected to methanol and crystal violet for fixation and staining. The number of migrated cells was counted under a microscope (IX71, Olympus, Tokyo, Japan).

### Western blot

U87 and U251 cells were lysed with the help of RIPA lysis buffer (Beyotime Biotechnology, China). Primary antibodies, including E-cadherin (ab1416), *N*-cadherin (ab18203), Vimentin (ab92547), β-catenin (ab32572), cyclin D1 (ab16663), c-myc (ab32072) and GAPDH (ab8245) were purchased from Abcam company (Abcam, Cambridge, UK). Blots were imaged by ECL detection reagents (Amersham Biosciences, Sweden).

### Microarray and KEGG pathway analysis

Total RNA extracted from glioblastoma tissues was washed by RNeasy mini kit (QIAGEN, Germany) under the instructions by manufacturers. RNA quality was thoroughly assessed by Qubit Fluorometer (Thermo Fisher Scientific, USA). Raw data were processed and subjected to KEGG pathway analysis.

### RNA immunoprecipitation (RIP) assay

Magna RNA immunoprecipitation (RIP) kit (Millipore, Billerica, USA) was used for conducting RIP assay in U87 and U251 cells. Magnetic beads containing Ago2 or IgG (negative control) antibodies were added into cell lysate which was preserved in RIP buffer before. Relative expression of SNHG29, miR-223-3p and CTNND1 was detected by RT-qPCR.

### Luciferase reporter assay

SNHG29-WT (or CTNND1-WT) and SNHG29-Mut (or CTNND1-Mut) were constructed into pmirGLO plasmids (Promega, Madison, USA). The constructed plasmids were co-transfected with miR-223-3p mimics or NC mimics into U87 and U251 cells for 48 h. Luciferase reporter assay system (Promega, Madison WI) was adopted to examine the relative luciferase activities.

### Statistical analysis

SPSS 17.0 statistics software (SPSS, Inc., Chicago, IL, USA) was responsible to statistical analysis. Data in graphs were shown as the mean ± standard deviation (SD) from experiments that were repeated at least 3 times. The group differences were separately analyzed by Student’s t test and one-way ANOVA test. A value of P less than 0.05 was regarded as statistically significant.

## Result

### SNHG29 accelerated cell proliferation, migration and EMT process with higher expression in glioblastoma cells

**Fig. 1:**
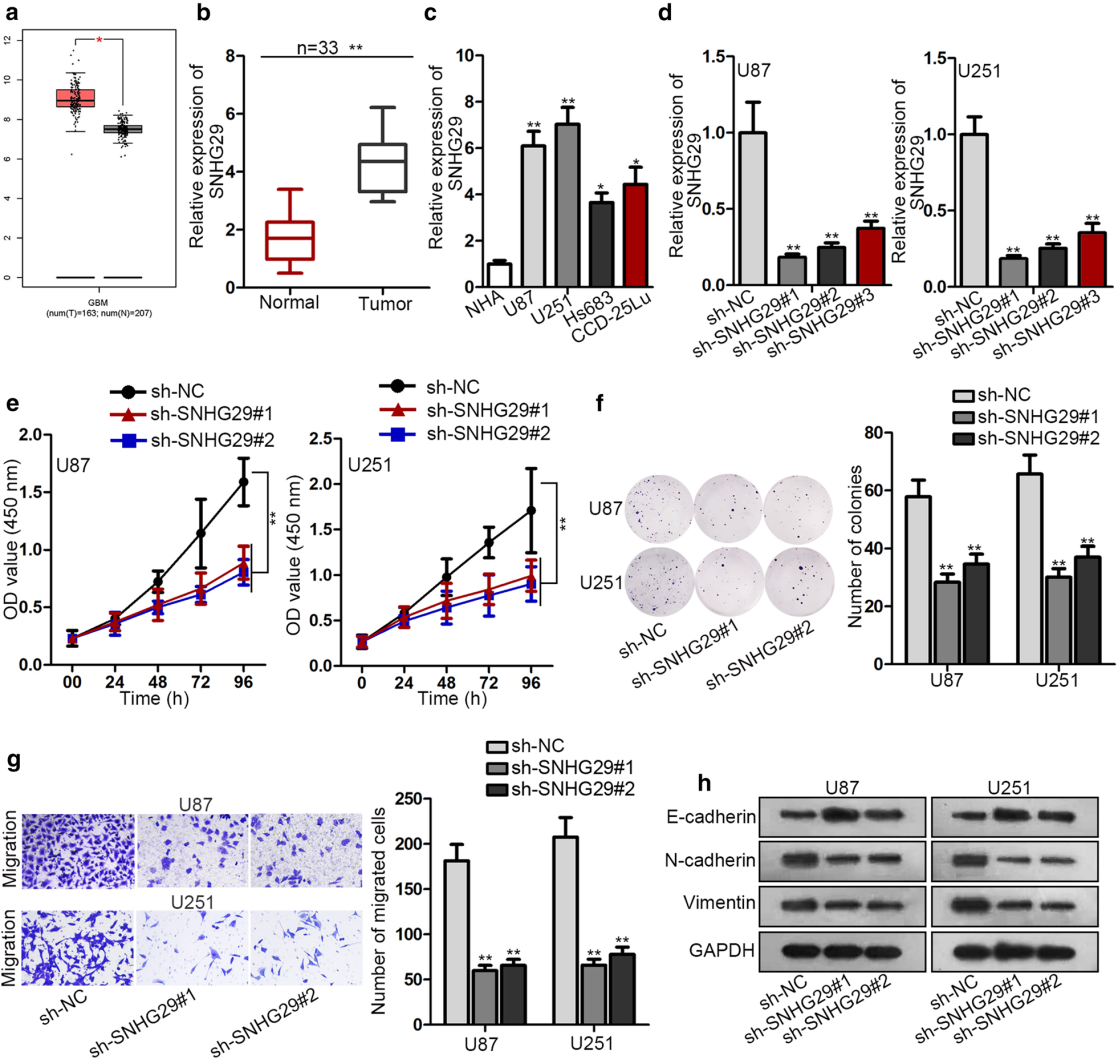
SNHG29 accelerated cell proliferation, migration and EMT process with higher expression in glioblastoma cells. **a** Data from GEPIA showed the expression status of SNHG29. **b**, **c** SNHG29 expression in tissues or cells was detected by RT-qPCR assay. **d** The knockdown efficacy of sh-SNHG29#1/2/3 was evaluated by RT-qPCR assay. **e**, **f** The proliferative ability was assessed by CCK-8 and colony formation assay. **g** Cell migration was explored by transwell assay. **h** The levels of EMT-related proteins was examined by western blot assay. *P < 0.05, **P < 0.01

SNHG29 (LRRC75A-AS1) has been reported to play an oncogenic role in osteosarcoma, the specific role of SNHG29 appealed to us a lot. According to GEPIA database (http://gepia.cancer-pku.cn/), SNHG29 expression was up-regulated in glioblastoma (Fig. 1a). Additionally, RT-qPCR assay demonstrated that glioblastoma tissues and cells displayed higher expression than normal tissues and cells (Fig. 1b, c). Afterwards, two groups of 33 patients were subjected to Kaplan–Meier analysis, the result indicated that SNHG29 expression had a negative association with the overall survival of glioblastoma patients (Additional file 1: Fig. S1A). High level of SNHG29 was found to be associated with tumor size and WHO stage of patients (Table 1). To determine the specific function of SNHG29, SNHG29 expression was dramatically reduced by transfection of sh-SNHG29#1, sh-SNHG29#2 or sh-SNHG29#3 (Fig. 1d), and sh-SNHG29#1 and sh-SNHG29#2 were selected for loss-of-function assays for their better knockdown efficiency. In loss-of-function assays, the proliferative capability of U87 and U251 cells was distinctly hampered by SNHG29 deficiency (Fig. 1e, f). Subsequently, the number of migrated U87 and U251 cells was also decreased by the knockdown of SNHG29 (Fig. 1g). In the end, upon transfecting sh-SNHG29#1 or sh-SNHG29#2 into U87 and U251 cells, the E-cadherin expression elevated whilst the expression of *N*-cadherin and Vimentin plummeted (Fig. 1h). In conclusion, higher expression of SNHG29 in glioblastoma cells accelerated cell proliferation, migration and EMT process.

**Table 1:** Correlation between SNHG29 expression and clinical features (n = 33)

Variable	SNHG29 Expression		*P* value
	Low	High	
Age			
< 50	10	12	0.721
≥ 50	6	5	
Gender			
Male	9	8	0.731
Female	7	9	
Tumor size			0.005^**^
< 5	12	4	
≥ 5	4	13	
WHO grade			0.01^*^
I–II	9	2	
III–IV	7	15	

Low/high by the sample median. Pearson χ^2^ test. ^**^P < 0.01, ^*^P < 0.05 was considered to be statistically significant

### SNHG29 directly interacted with miR-223-3p in glioblastoma

**Fig. 2:**
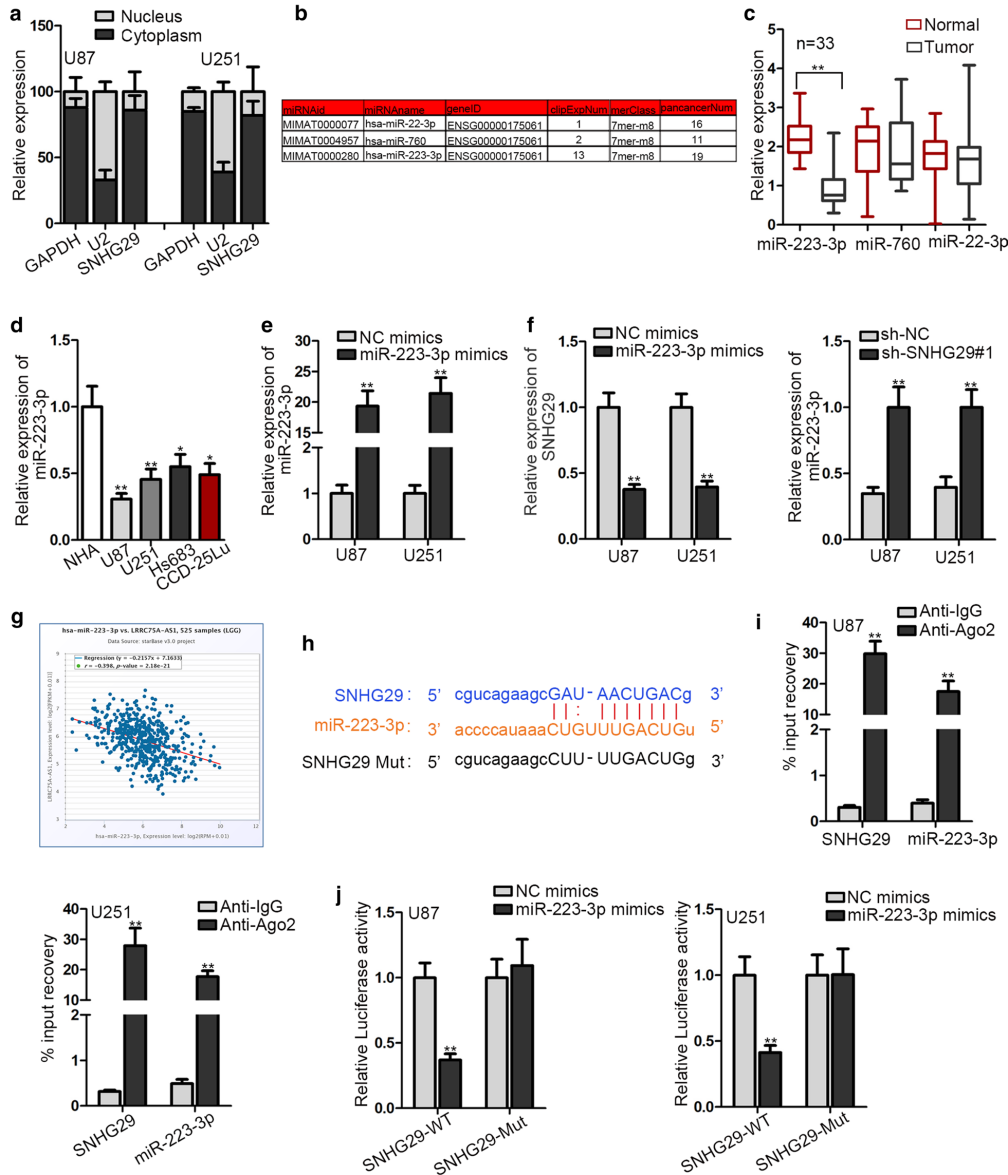
SNHG29 directly interacted with miR-223-3p in glioblastoma. **a** Nuclear-cytoplasmic fractionation was conducted to locate SNHG29. **b** The predicted miRNAs were shown. **c** The predicted miRNAs expression in glioblastoma tissues was measured by RT-qPCR. **d**–**f** The expression of SNHG29 or miR-223-3p was monitored by RT-qPCR. **g** The correlation between SNHG29 and miR-223-3p was depicted. **h** The binding site of miR-223-3p on SNHG29 was shown. **i**, **j** The binding capacity between SNHG29 and miR-223-3p was testified by RIP and luciferase reporter assays. *P < 0.05, **P < 0.01

Mechanistically, numerous researches have proposed a ceRNA pattern that lncRNA regulate tumor progression by sponging miRNA and target mRNA. Thus, we hypothesized SNHG29 also work in this mechanism. Nuclear-cytoplasmic fractionation illustrated that the majority of SNHG29 distributed in cytoplasm of U87 and U251 cells (Fig. 2a). StarBase (http://starbase.sysu.edu.cn) website was adopted to predict and filter potential miRNAs. As shown in Fig. 2b, 3 miRNAs were selected after screening (conditions: strict stringency of CLIP Data; low stringency of Degradome Data; 10 cancer types of Pan-Caner). To further determine which miRNA interact with SNHG29 in glioblastoma the result of RT-qPCR assay revealed the downregulation of miR-223-3p expression in glioblastoma tissues compared with adjacent normal tissues (Fig. 2c). Additionally, relative to normal human astrocyte (NHA) cells, glioblastoma cells presented lower miR-223-3p expression (Fig. 2d). Then, miR-223-3p expression was markedly increased with the upregulation of miR-223-3p (Fig. 2e). Based on Fig. 2f, miR-223-3p overexpression dropped SNHG29 expression and SNHG29 depletion enhanced miR-223-3p expression. Over and above that, Data from starBase displayed a negative association of SNHG29 and miR-223-3p in glioblastoma (Fig. 2g). To confirm the binding capacity between SNHG29 and miR-223-3p, the binding sequences between them were predicted from starBase (Fig. 2h). RIP assay uncovered that both SNHG29 and miR-223-3p had a high enrichment in Ago2 antibody, implying the co-existence of SNHG29 and miR-223-3p in RNA-induced silencing complex (RISC) (Fig. 2i). Luciferase reporter assay revealed that the luciferase activity of pmirGLO-SNHG29-WT was prominently weakened after increasing of miR-223-3p expression (Fig. 2j). All the experimental results testified the direct interaction of SNHG29 with miR-223-3p in glioblastoma.

### CTNND1 served as a target gene of miR-223-3p in glioblastoma

**Fig. 3:**
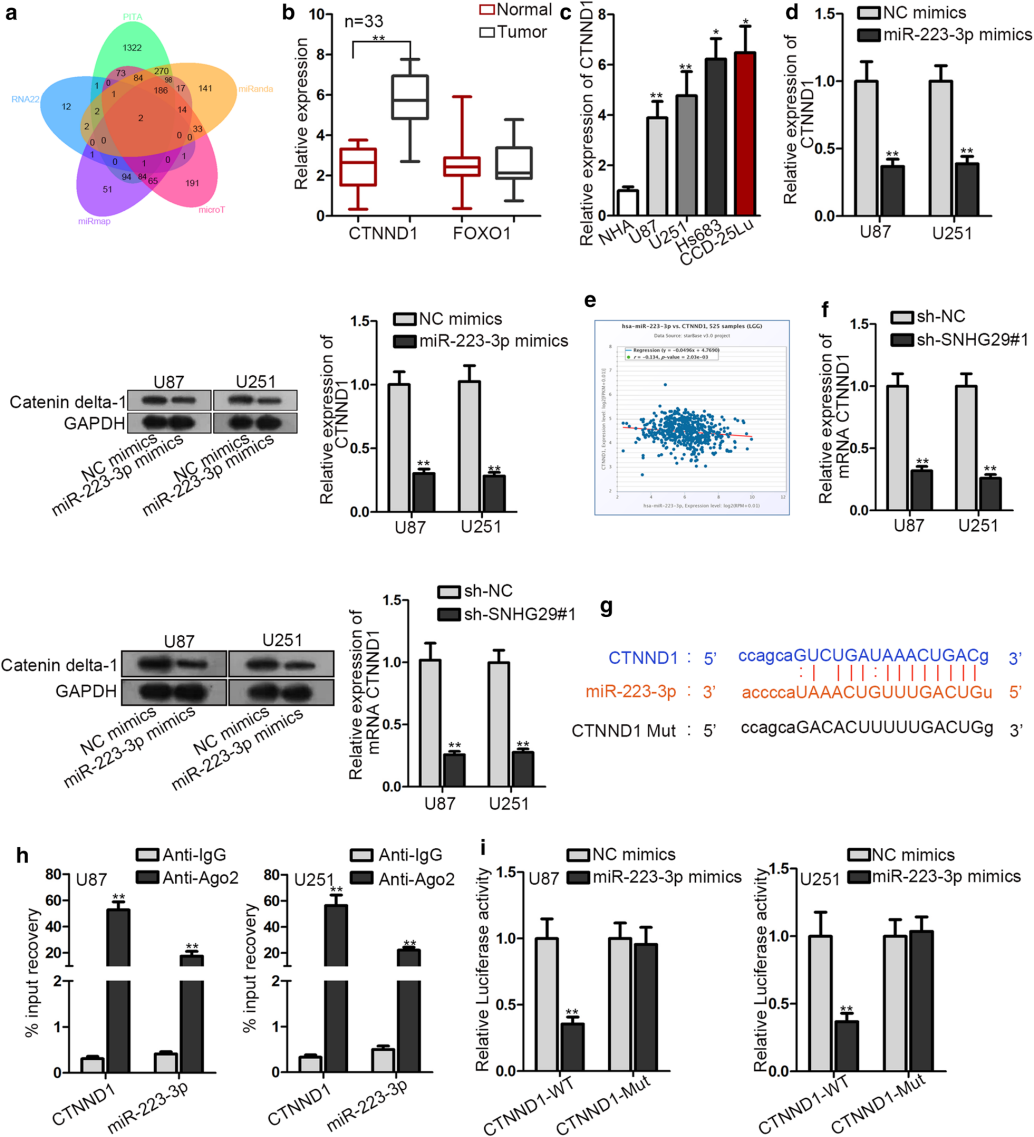
CTNND1 served as a target gene of miR-223-3p in glioblastoma. **a** The Venn diagram described the result of screening. **b**, **c** The expression of CTNND1 or FOXO1 was tested by RT-qPCR. **d** The detection of CTNND1 mRNA expression and catenin delta-1 protein expression was carried out in RT-qPCR and western blot assay. **e** Data from starBase indicated a negative correlation between miR-223-3p and CTNND1. **f** The examination of CTNND1 mRNA expression and catenin delta-1 protein expression was carried out in RT-qPCR and western blot assay. **g** The binding site of miR-223-3p on CTNND1 was predicted by starBase. **h**, **i** The interaction between miR-223-3p and CTNND1 was confirmed by RIP and luciferase reporter assays. **P < 0.01

To seek the target gene of miR-223-3p, two mRNAs were conjectured to bind with miR-223-3p according to PITA, RNA22, miRmap, microT and miRanda (Fig. 3a). Compared with control, CTNND1 expression was up-regulated in glioblastoma tissues whereas FOXO1 indicated no differential expression (Fig. 3b). Meanwhile, CTNND1 expression was also up-regulated in glioblastoma cells (Fig. 3c). The transfection of miR-223-3p mimics caused an obvious decline of CTNND1 expression and catenin delta-1 protein expression (Fig. 3d). The negative correlation between miR-223-3p and CTNND1 was shown in Fig. 3e. Moreover, the CTNND1 expression and catenin delta-1 protein expression was lowered by SNHG29 repression (Fig. 3f). Then, the binding site of miR-223-3p on CTNND1 3′UTR was predicted by starBase (Fig. 3g). RIP assay illustrated the abundance of miR-223-3p and CTNND1 in anti-Ago2 instead of anti-IgG, which means both miR-223-3p and CTNND1 were co-immunoprecipitated by Ago2 antibody (Fig. 3h). Luciferase reporter assay manifested that miR-223-3p overexpression resulted in an attenuation of luciferase activity of pmirGLO- CTNND1-WT (Fig. 3i). Taken together, CTNND1 served as the downstream target of miR-223-3p in glioblastoma.

### SNHG29 promoted glioblastoma progression by regulating CTNND1 expression

**Fig. 4:**
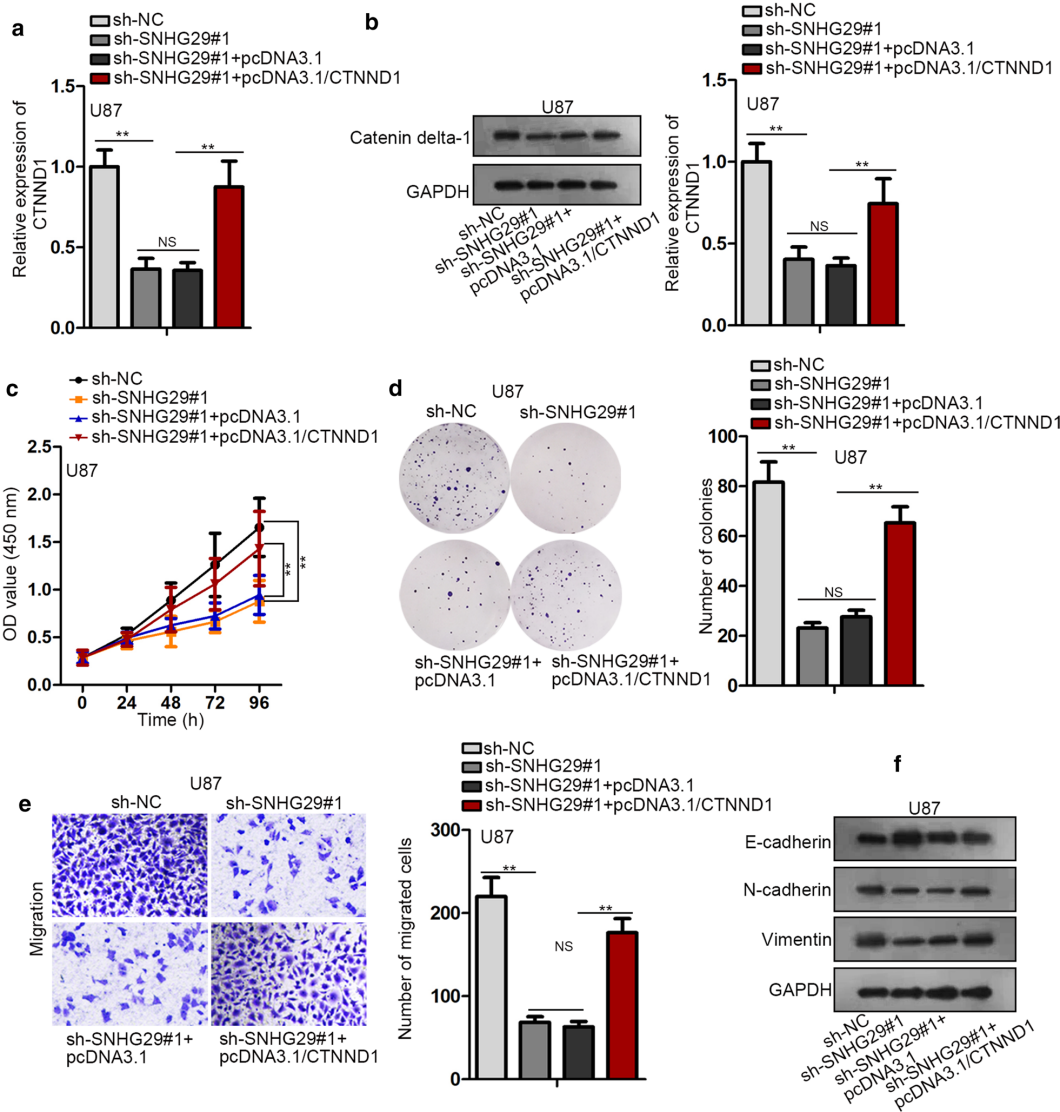
SNHG29 promotes glioblastoma progression by regulating CTNND1 expression. **a**, **b** The measurement of CTNND1 mRNA expression and catenin delta-1 protein expression was conducted in RT-qPCR and western blot assay. **c**–**e** The evaluation of cell proliferative and migratory ability was implemented in CCK-8, colony formation and transwell assays. **f** The examination of proteins expression of E-cadherin, N-cadherin and vimentin was operated in western blot. *P < 0.05, **P < 0.01

Rescue assays were implemented to fully proof SNHG29 exert an oncogenic role through miR-223-3p/CTNND1 axis. To begin with, the inhibition of SNHG29 silence on CTNND1 mRNA and catenin delta-1 protein levels was abrogated by CTNND1 overexpression (Fig. 4a, b). Then, the inhibitory effect of SNHG29 knockdown on the proliferative ability of glioblastoma cells was abolished by transfection pcDNA3.1/CTNND1 into U87 and U251 cells (Fig. 4c, d). By the same token, CTNND1 amplification recovered the reduction of migrated cells caused by SNHG29 repression (Fig. 4e). Eventually, sh-SNHG29#1-mediated the enhancement of E-cadherin level, the reduction of *N*-cadherin and vimentin were counteracted by CTNND1 amplification (Fig. 4f). Collectively, SNHG29 promoted glioblastoma progression by regulating CTNND1 expression.

### SNHG29 up-regulated CTNND1 expression to promote glioblastoma progression via Wnt/β-catenin signaling pathway

**Fig. 5:**
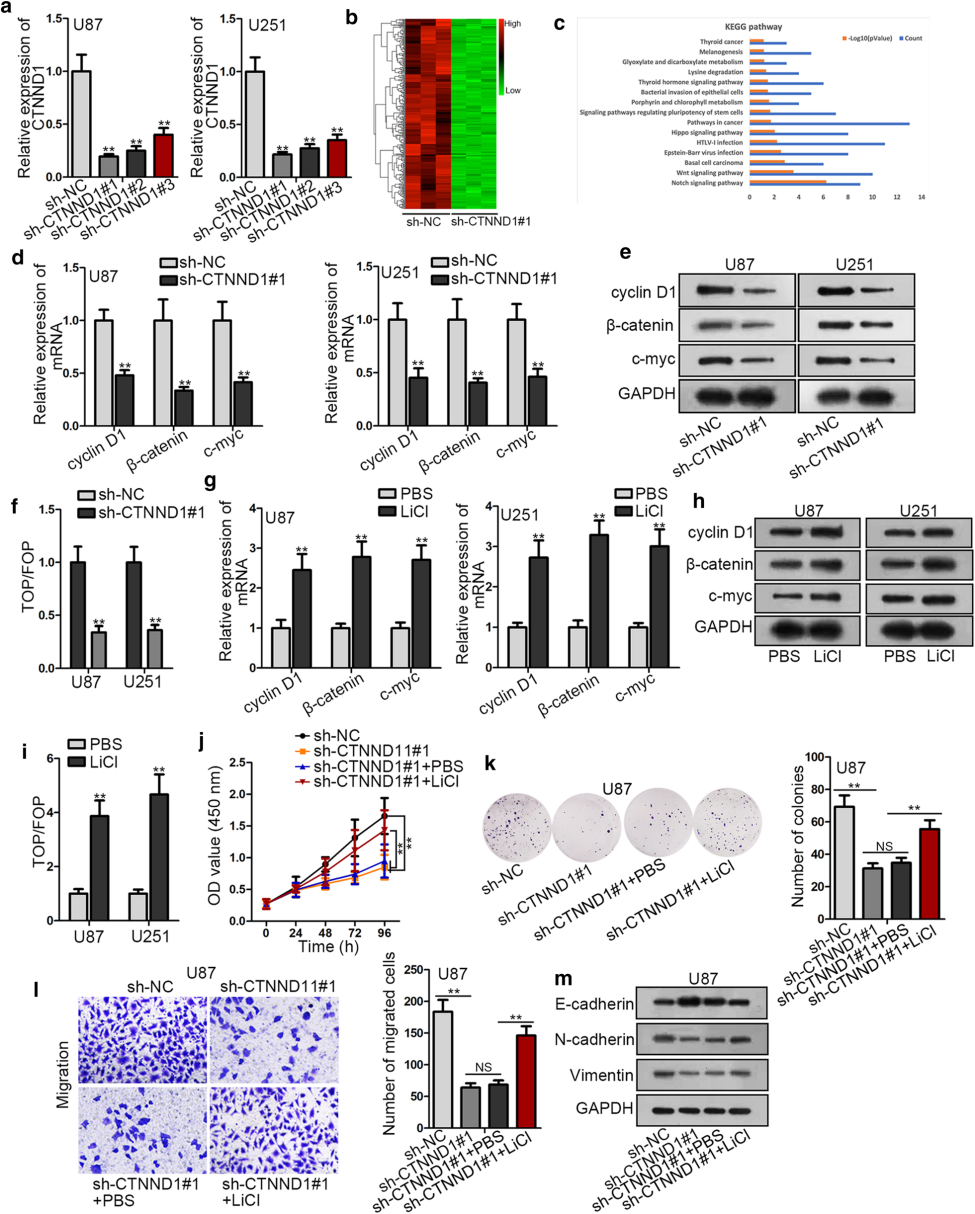
SNHG29 up-regulated CTNND1 expression to promote glioblastoma progression via Wnt/β-catenin signaling pathway. **a** The knockdown efficiency was validated by RT-qPCR. **b** The genes expression was researched by microarray. **c** The down-regulated genes were analyzed by KEGG analysis. **d**, **e** The mRNA and protein expression of cyclin D1, β-catenin and c-myc was determined by RT-qPCR and western blot assays. **f** TOP-FLASH assay was used to examine the effect of SNHG29 on the activity of Wnt/β-catenin signaling pathway. **g**, **h** The assessment of mRNA and protein expression of cyclin D1, β-catenin and c-myc was conducted in RT-qPCR and western blot assays. **i** TOP-FLASH assay was used to examine the effect of LiCl on the activity of Wnt/β-catenin signaling pathway. **j**–**l** The investigation of cell proliferation and migration was performed by CCK-8, colony formation and transwell assays. **m** The examination of proteins expression related to EMT process was operated in western blot. *P < 0.05, **P < 0.01. *NS* not significant

Wnt/β-catenin signaling pathway is known as modulators for the proliferation and metastasis of tumors. Therefore, we speculated that CTNND1 mediated the oncogenic role of SNHG29 through Wnt/β-catenin signaling pathway. To confirm our hypothesis, a series of experiments were carried out. CTNND1 expression was obviously decreased by transfection of sh-CTNND1#1, sh-CTNND1#2 or sh-CTNND1#3 (Fig. 5a). Particularly, sh-CTNND1#1 possessed a better knockdown efficiency. As depicted in Fig. 5b, in the context of CTNND1 absence, down-regulated genes were shown in heatmap. Through KEGG analysis, we discovered that these down-regulated genes were enriched in Wnt/β-catenin signaling pathway (Fig. 5c). Besides, the mRNA and protein expression of Wnt/β-catenin signaling pathway markers including cyclin D1, β-catenin and c-myc was conspicuously dwindled by CTNND1 restraint (Fig. 5d, e). On top of that, luciferase activity of TOP was significantly lessened by CTNND1 knockdown (Fig. 5f). On the contrary, the addition of LiCl (an agonist of Wnt/β-catenin signaling pathway) greatly raised the mRNA and protein expression of cyclin D1, β-catenin and c-myc (Fig. 5g, h). The luciferase activity of TOP was also elevated by LiCl addition (Fig. 5i). Then, the alleviation of cell proliferation induced by sh-CTNND1#1 was countervailed by the treatment of LiCl (Fig. 5j, k). Afterwards, the LiCl treatment also retarded the inhibited impacts of CTNND1 deficiency on cell migration (Fig. 5l). The transfection of sh-CTNND1#1 multiplied E-cadherin but diminished *N*-cadherin and vimentin expression, and these influences were then destroyed by LiCl addition (Fig. 5m). All in all, SNHG29 regulates miR-223-3p/CTNND1 axis to promote glioblastoma progression via Wnt/β-catenin signaling pathway.

## Discussion

Glioblastoma is one of the most lethal types of tumors in the central nervous system [12]. Surgery section, chemotherapy and radiotherapy were widely adopted to treat patients with malignant brain tumor whereas the result of these methods did not improve patient’s condition [13]. The pathology and progression of glioblastoma was associated with both genetic and epigenetic changes [14]. Alterations of molecules also have been reported to be associated with the recurrence of patients in primary glioblastoma [15]. Thus, better understanding and further exploration of underlying mechanism of glioblastoma was urgently needed.

LncRNAs is a category of non-coding RNAs, playing an important part in pathological and physiological aspects [16, 17]. The dysregulation of lncRNAs was closely related to cellular processes of tumors. Up-egulation of lncRNA LINC00174 promotes cell proliferation to facilitate colorectal carcinoma progression via miR-1910-3p/TAZ axis [18]. Additionally, up-regulation of SNHG14 boosts cell migration and invasion in renal cell carcinoma [19]. Likewise, glioblastoma tissues and cells also displayed higher SMHG29 expression than normal tissues and cells. Moreover, knockdown of SNHG29 limited glioblastoma cell proliferation, migration and EMT process.

Mechanistically, LncRNAs have been proved by abundant explorations to serve as a ceRNA to regulate tumor progression [20–22]. Based on the theory of ceRNA pattern, we speculated that SNHG29 also functioned in this pattern. In our research, miR-223-3p expression was validated to combine with SHNG29 after prediction and screening. The expression of miR-223-3p was negatively correlated with SNHG29 expression. Furthermore, CTNND1 was then proved to serve as a target gene of miR-223-3p after the prediction of starBase and screening. Additionally, CTNND1 was negatively correlated with miR-223-3p. The rescue assays suggested that CTNND1 overexpression restored the inhibitory influence of SNHG29 knockdown on cell proliferation, migration and EMT process.

The Wnt/β-catenin signaling pathway has been identified to be closely associated with the regulation of many cellular events (proliferation, differentiation, migration, or EMT process) through modulating the ability of β-catenin protein [23–25]. Recent studies demonstrated that some lncRNAs could affect the Wnt/β-catenin signaling pathway in multiple cancers [26–28] In the current research, the markers expression of Wnt/β-catenin signaling pathway including β-catenin, c-myc and cyclin D1 was respectively decreased by CTNND1 suppression and increased by addition of LiCl. At last, the retraining effect on cell proliferation, migration and EMT process of Wnt/β-catenin signaling pathway inactivation caused by CTNND1 repression was abolished by LiCl addition. Collectively, this study analyzed the association between ANHG29 expression and the overall survival of glioblastoma patients, indicating the prognostic potential of SNHG29 in glioblastoma patients. Further clinical study will be made in our future study. Lack of animal study and absent of mechanism investigation on SNHG29 upstream are pitfalls of our current study, we will investigate more deep mechanism of this molecular pathway in future study.

## Conclusion

This research was the first time to investigate the function and mechanism of SNHG29 in glioblastoma and we verified that SNHG29 regulates miR-223-3p/CTNND1 axis to promote glioblastoma progression via Wnt/β-catenin signaling pathway. However, this was just the initial exploration of SNHG29 in glioblastoma and other mechanisms of SNHG29 in glioblastoma remained to be explored in the future.

## Supplementary information

**Additional file 1: Figure S1.** (A) Overall survival analysis of glioblastoma patients with high or low level of SNHG29. *P = 0.015.

## Data Availability

Research data can be shared if necessary.

## Abbreviation

LncRNA: long noncoding RNA
SNHG29: small nucleolar RNA host gene 29
CeRNA: competitive endogenous RNA
mRNA: messenger RNA
MiRNA: microRNA
NHA: normal human astrocyte
DMEM: Dulbecco’s Modified Eagle Medium
FBS: fetal bovine serum
ShRNAs: short hairpin RNAs
RT-qPCR: real-time reverse-transcription polymerase chain reaction
PBS: phosphate buffer saline
GAPDH: glyceraldehyde 3‐phosphate dehydrogenase
CCK-8: the cell counting kit-8
RIPA: radio immunoprecipitation assay
PVDF: polyvinylidene fluoride
SDS–PAGE: sodium dodecyl sulfate–PAGE
KEGG: Kyoto Encyclopedia of Genes and Genomes
3′ UTR: 3′ untranslated region
SNHG29-WT: wild-type SNHG29 reporter
SNHG29-Mut: mutant-type SNHG29 reporter
CTNND1-WT: wild-type CTNND1
CTNND1-Mut: CTNND1 circ-HIPK2
RIP assay: RNA immunoprecipitation assay
SPSS: Statistical Package for Social Sciences
ANOVA: analysis of variance
