# Supplementary figures and images for: SNHG29 regulates miR-223-3p/CTNND1 axis to promote glioblastoma progression via Wnt/β-catenin signaling pathway
Source: Cancer Cell Int. 2019 Dec 19;19:345. doi: 10.1186/s12935-019-1057-x (PMC6924063; doi:10.1186/s12935-019-1057-x)

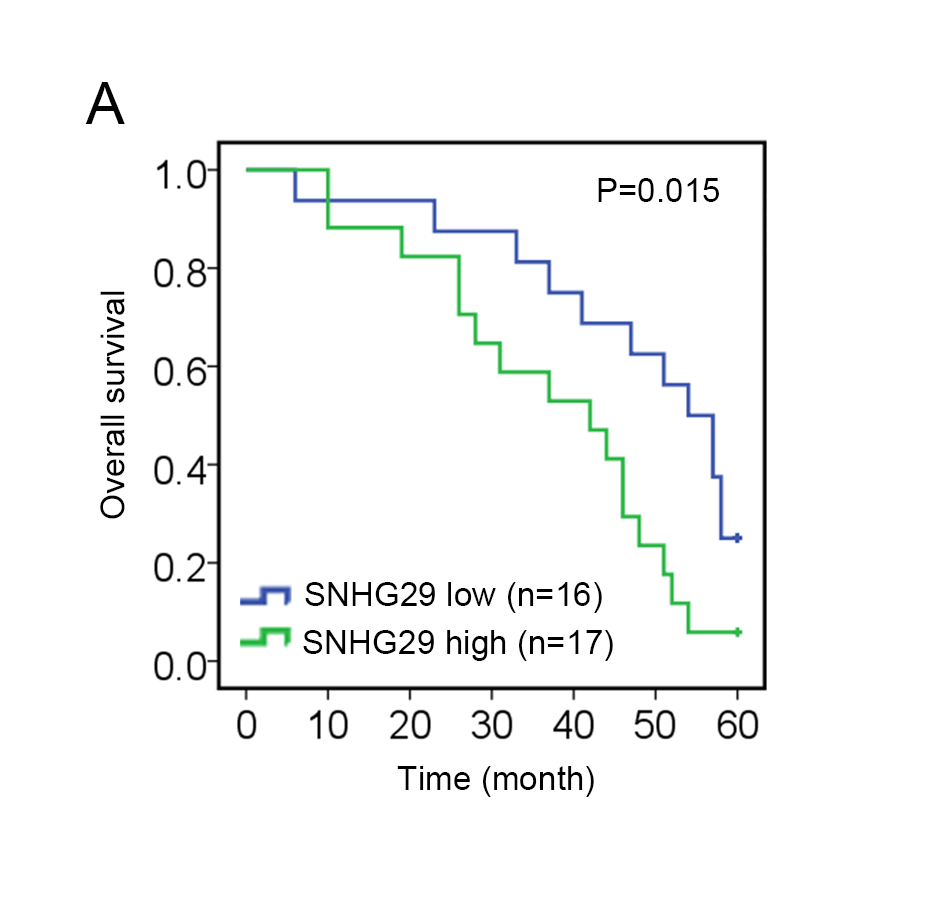

Supplement: Supplementary file 1 — Additional file 1: Figure S1. (A) Overall survival analysis of glioblastoma patients with high or low level of SNHG29. *P = 0.015. [file 12935_2019_1057_MOESM1_ESM.tif]
